# Prognostication in emergency room patients: comparing ultrasensitive and contemporary quantification of cardiac troponin levels below the 99th percentile

**DOI:** 10.3389/fcvm.2024.1450619

**Published:** 2025-01-13

**Authors:** Anna Carrasquer, Germán Cediel, Alma Gómez-Sanz, Óscar M. Peiró, Isabel Fort-Gallifa, Alfredo Bardaji, Jose Luis Ferreiro

**Affiliations:** ^1^ Department of Cardiology, Joan XXIII University Hospital, Tarragona, Spain; ^2^ Pere Virgili Health Research Institute, Rovira I Virgili University, Tarragona, Spain; ^3^ Department of Medicine and Surgery, Rovira I Virgili University, Tarragona, Spain; ^4^ Clinical Analysis Service, University Hospital of Tarragona Joan XXIII, Tarragona, Spain

**Keywords:** AMI, acute myocardial infarction, cTn, cardiac troponin 2, troponin, prognosis, mortality, heart failure

## Abstract

**Introduction:**

Cardiac troponin levels below the 99th percentile improve the predictive efficacy for cardiovascular events when associated with relevant clinical variables. However, whether ultra-sensitive analytical methods improve this predictive efficacy over less sensitive or contemporary analytical methods remains unknown.

**Methods:**

This retrospective observational study involved consecutive patients who presented to the emergency department for suspected acute coronary syndrome and underwent measurement of ultra-sensitive cardiac troponin I (Singulex) and contemporary cardiac troponin I (Siemens) with levels below the 99th percentile. The clinical characteristics of these patients were analysed, and the efficacy of both analytical methods for predicting cardiovascular events over a 4-year follow-up period was compared.

**Results:**

In total, 838 patients were analysed (mean age, 62.9 ± 16.6 years; 42.2% women). Their cumulative incidence of the composite cardiovascular event (death, readmission for myocardial infarction, and readmission for heart failure) was 25.9% over the 4-year follow-up. Both Singulex cardiac troponin I (analysed by quartiles) and Siemens cardiac troponin (analysed as detectable/undetectable) improved the predictive efficacy for the combined event over clinical variables [Harrell's C-index (95% confidence interval): 0.77 (0.74–0.80) vs. 0.79 (0.76–0.81) and 0.77 (0.74–0.80) vs. 0.78 (0.75–0.81), respectively; *p* = 0.018]. However, there were no statistically significant difference between the two predictive models that included the aforementioned troponin assays.

**Conclusions:**

Detectable levels of cardiac troponin using a contemporary analytical method or those near the 99th percentile using an ultra-sensitive analytical method improve the predictive efficacy for cardiovascular events, with no differences between the two methods

## 1 Introduction

The quantification of cardiac troponin (cTn) in patients with chest pain or suspected acute coronary syndrome (ACS) allows for the confirmation or exclusion of acute myocardial infarction (AMI) with acceptable safety (1). The continuous evolution in analytical methods for quantifying cardiac troponin T (cTnT) or cardiac troponin I (cTnI) has increased the positive and negative predictive values for the diagnosis of AMI in the presence of elevated (above the 99th percentile) or non-elevated (below the 99th percentile) cTn levels (2). An elevated cTn level in a patient with clinical evidence of myocardial ischaemia enables the diagnosis of AMI (3). The cTn level is also a significant predictor of medium- and long-term cardiovascular events both in patients with AMI and in patients with acute or chronic myocardial injury of non-ischaemic causes (4–7).

In recent years, there has been growing interest in the predictive value of cTn below the 99th percentile not only for ruling out myocardial infarction but also for predicting future cardiovascular events (8–11). Using contemporary or high-sensitivity analytical methods, cases of non-elevated cTn can usually be categorised into those with undetectable and detectable cTn. However, the proportion of patients with undetectable cTn—more than 50% with contemporary methods and less than 50% with high-sensitivity methods (12)—does not allow for analysis of the entire spectrum of cTn levels below the 99th percentile. The Singulex Sgx Clarity cTnI Assay (Singulex Inc., Alameda, CA, USA), based on Single Molecule Counting (SMC®) technology, is an ultrasensitive immunoassay that uses single-molecule fluorescence detection for cTnI quantification. With this assay, which shows excellent analytical performance, cTnI can be detected in practically 100% of samples from normal patients (13).

We hypothesised that by using this ultrasensitive method, we can accurately stratify the risk of medium- to long-term cardiovascular events in patients with cTnI below the 99th percentile, improving upon the prediction obtained with clinical variables. In the present study, we analysed the predictive value of different cTnI Sgx values for cardiovascular events in comparison to the contemporary cTnI analysis method (described below) in patients with cTnI below the 99th percentile.

## 2 Methods

### 2.1 Study design

This retrospective observational cohort study involved all patients admitted to the emergency department of a tertiary university hospital for chest pain or symptoms compatible with ACS from February 2018 to February 2019. All patients had at least one cTnI measurement requested at the discretion of the treating physician, following the local protocol for the care of such patients in the emergency department. Patients were identified from the lists of urgent analytical determinations carried out by the centre's laboratory, which quantified the number of troponin tests for each patient and recorded the maximum detected value.

### 2.2 Assay

The ADVIA Centaur TnI-Ultra Immunoassay (Siemens Healthineers, Erlangen, Germany) was performed as the contemporary cTnI analysis method. The lower detection limit of 6 ng/L was established by the manufacturer. The reference limit for a positive cTnI-Ultra test was >39 ng/L, corresponding to the 99th percentile of a reference control group, with analytical imprecision expressed by a coefficient of variation below 10%.

The Singulex Sgx Clarity system mentioned in the Introduction is a paramagnetic microparticle-based immunoassay powered by SMC® technology that uses single-photon fluorescence detection for analyte quantitation. This assay employs a 2 × 2 pair of monoclonal antibodies that recognise epitopes in the central region and at both ends of the cTnI molecule. The cut-off point used to consider an elevated cTnI Sgx level was 8.67 pg/ml, corresponding to the 99th percentile, determined with a coefficient of variation below 5.7% (13).

### 2.3 Study population

**Figure 1:**
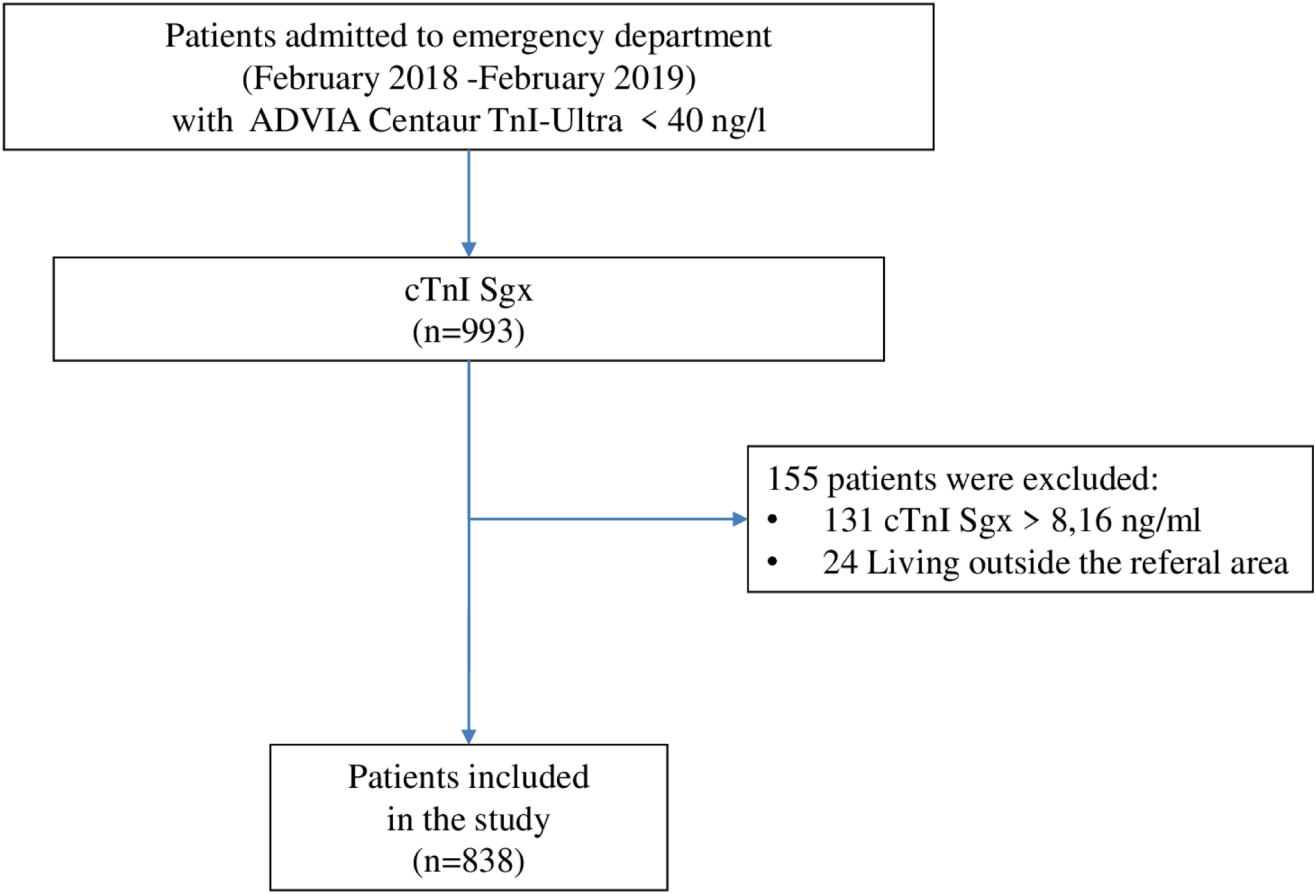
Flow diagram of patients.

In patients with Siemens cTnI-Ultra levels below the 99th percentile, the cTnI Sgx level was determined. Among these patients, those with levels below the 99th percentile were selected. Thus, patients with both cTnI-Ultra and cTnI Sgx levels below the 99th percentile were included in the analysis. The exclusion criteria were an age of <18 years and the inability to be followed up either because of residence outside our direct reference area or because of the absence of clinical follow-up data (Figure 1). Demographic data, medical history, clinical and analytical variables, electrocardiographic data, and examinations performed on all patients included in the study were collected. The Charlson Index (CI) score was calculated in all patients (14). Whether the patient was admitted to the hospital or discharged directly from the emergency department was recorded and the final diagnosis was established at the treating physician's discretion.

### 2.4 Follow-up events

The primary outcome of the study was the combined events of total mortality or readmission for myocardial infarction or heart failure (major cardiovascular events) at the 4-year follow-up. The secondary outcomes were total mortality and the readmission rates for heart failure or myocardial infarction. The events that occurred during follow-up were obtained from the patients’ electronic medical records and death records.

### 2.5 Statistical analysis

Categorical variables are expressed as number and percentage. Continuous variables are expressed as mean ± standard deviation or median and interquartile range depending on whether their normal or non-normal distribution. Then, normality of the data were assessed using Q-Q plots. For continuous variables following a normal distribution, the ANOVA test was used to assess in-between differences across groups whereas for not normally distributed continuous variables, the Kruskal Wallis test. Bonferroni correction was used in the four-group comparisons. Categorical variables were compared across the groups with Chi-Squared Test. The associations between quartiles of cTnI Sgx levels and detectable cTnI-Ultra levels with the clinical endpoints were examined using univariable and multivariable Cox regression analyses. The following variables were incorporated into the regression models: age, sex, history of myocardial infarction, heart failure, cerebrovascular disease, hypertension, diabetes mellitus, and glomerular filtration rate. To analyse readmission for heart failure or AMI, a competing risk strategy was adopted using the Fine and Gray method, considering death as a competing event. Results are expressed as hazard ratio with corresponding 95% confidence interval. Adjusted survival curves were plotted. Harrell's C statistic was used to assess the discrimination benefit of adding the cTnI Sgx level or detectable cTnI-Ultra level to the clinical model. The difference between two Harreĺs C statistics have been calculated using the -somersd—and -lincom- STATA commands. This package provides the confidence interval of the difference between the 2 estimations. Statistical analyses were performed using STATA version 13.0 (StataCorp, College Station, TX, USA). A two-tailed *P*-value of <0.05 was considered statistically significant.

The local ethics committee approved this study.

## 3 Results

### 3.1 Patient population and follow-up

Table 1 shows the demographic data, risk factors, and major cardiovascular history of the general study population (*n* = 838). The patients’ mean age was 62.9 ± 16.6 years, and 42.2% were women. Admission was decided for 22.7% of patients, and hospital mortality reached 1%. The main diagnoses were chest pain (more than one-third of patients), followed by tachyarrhythmias and heart failure (Table 2). The incidence of combined major cardiovascular events was 25.9% (*n* = 217) at the 4-year follow-up. The total mortality, readmission for myocardial infarction, and readmission for heart failure rates were 16.6% (*n* = 139), 4.6% (*n* = 39), and 9.3% (*n* = 78), respectively, at the 4-year follow-up.

**Table 1:** Baseline characteristics of patients enrolled in the study.

	Total (*n* = 838)
Demographic variables	
Age, years	62.9 ± 16.6
Female sex	354 (42.2)
Clinical history	
Arterial hypertension	452 (53.9)
Diabetes	175 (20.9)
Current or previous smoker	134 (16.0)
Prior MI	172 (20.5)
Congestive heart failure	47 (5.6)
Peripheral arterial disease	38 (4.5)
Stroke or TIA	47 (5.6)
COPD	155 (18.5)
Charlson index	2 (0–2)
Symptoms	
Chest pain	475 (56.7)
Dyspnoea	44 (21.0)
Syncope	10 (4.8)
Other symptoms	66 (31.4)
Vital signs	
SPB, mmHg	136.1 ± 24.9
DBP, mmHg	79.4 ± 14.9
Heart rate, bpm	81.2 ± 21.2
Electrocardiogram	
Atrial fibrillation	91 (10.9)
LBBB or RBBB	78 (9.3)
Negative T wave	39 (4.7)
ST-depression	5 (0.6)
Laboratory tests	
eGFR, ml/min/1.73 m^2^	92 ± 34.2
Haemoglobin, g/dl	13 ± 1.9
cTnI Sgx, ng/L	5.3 ± 1.3
Hospitalisation	190 (22.7)
In-hospital mortality	5 (1.0)

Data are presented as mean ± standard deviation, *n* (%), or median (interquartile range).

Q, quartile; MI, myocardial infarction; TIA, transient ischaemic attack; COPD, chronic obstructive pulmonary disease; SBP, systolic blood pressure; DBP, diastolic blood pressure; LBBB, left bundle branch block; RBBB, right bundle branch block; eGFR, estimated glomerular filtration rate (calculated by Modification of Diet in Renal Disease Study equation); cTnI Sgx, cardiac troponin I Singulex.

**Table 2:** Main diagnoses at patient discharge among the patients analyzed.

	Total (*n* = 838)
Chest pain	311 (37.1)
Congestive heart failure	44 (5.3)
Tachyarrhythmia	55 (6.7)
Respiratory infection	64 (7.6)
Gastrointestinal bleeding	4 (0.5)
Other gastrointestinal pathology	48 (5.7)
Syncope	12 (1.4)
Other infections	13 (1.6)
Sepsis	1 (0.2)
Bradycardia	5 (0.6)
Anaemia	5 (0.6)
Neurological disease	10 (1.2)
Neoplasia	3 (0.4)
Aortic disease	2 (0.2)
Hypertensive crisis	15 (1.8)
Renal failure	2 (0.2)
Other diagnoses	164 (19.6)

Data are presented as *n* (%).

The total study population was divided into four groups based on the quartiles of the cTnI Sgx levels, the mean of which was 5.3 ± 1.3 ng/ml. Supplementary Table S1 presents the clinical characteristics of the analysed groups. Patients with cTn I Sgx levels in the highest quartile were older, had more cardiovascular risk factors (diabetes, smoking, and hypertension), and had more comorbidities (history of heart failure, history of myocardial infarction, cerebrovascular disease, chronic lung disease, and renal failure) and a higher CI score. The main symptom was chest pain, followed by dyspnoea. On electrocardiography, higher proportions of atrial fibrillation and intraventricular conduction disorders were detected, an other electrocardiography alterations like negative T wave or ST-depression were less frequency. The analysis also revealed higher rates of anaemia and worse glomerular filtration rates in this quartile. Hospital admission was decided for 33.3% of patients in the highest quartile compared with the other quartiles. Hospital mortality reached 1.4% in the highest quartile, but this rate did not reach statistical significance. Supplementary Table S2 shows the main diagnoses by quartiles of cTnI Sgx levels. The incidence rates of the combined events, as well as total mortality, readmission for myocardial infarction, and readmission for heart failure at 4 years of follow-up, increased significantly according to the quartile of cTnI Sgx levels (Supplementary Table S3).

In total, 344 (41%) patients had detectable cTnI-Ultra levels below the 99th percentile. The remaining 494 (59%) had undetectable cTnI levels. Supplementary Table S4 presents the clinical characteristics of the analysed patients. Patients with detectable cTnI levels were older, had more cardiovascular risk factors (diabetes and hypertension), had more cardiovascular comorbidities and a higher CI score. This clinical risk profile was similar to that of patients with cTnI Sgx levels in the highest quartile. The main symptom in these patients was also chest pain, followed by dyspnoea. Patients with detectable cTnI levels had small but statistically significant differences in their electrocardiographic abnormalities (higher proportions of atrial fibrillation and intraventricular conduction disorders) and analytical parameters (higher degree of anaemia and worse glomerular filtration rates). Hospital admission was decided for 13.1% of patients, and the hospital mortality rate was 0.5% but did not reach statistical significance. Supplementary Table S5 shows the main diagnoses in patients with detectable and undetectable cTnI-Ultra levels. The main diagnosis in these patients was also chest pain, which occurred in 11.3%, followed by heart failure in 3.5%; both were statistically significant. The incidence rate of the combined events was higher in patients with detectable than undetectable cTnI levels, as was the incidence of total death, admission for myocardial infarction, and admission for heart failure (Supplementary Table S6).

### 3.2 Predictive capacity of cTnI (cTnI Sgx and cTnI-ultra levels)

**Figure 2:**
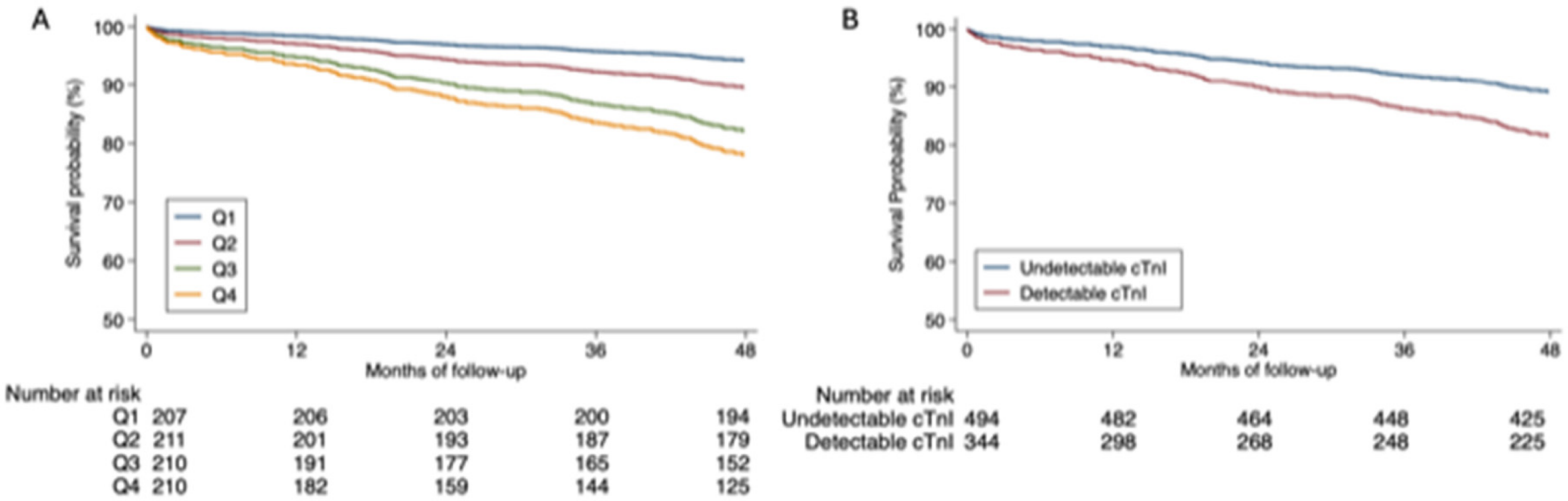
Cumulative survival. **(A)** Cumulative survival according to quartiles of cardiac troponin I Singulex. **(B)** Cumulative survival according to detectable cardiac troponin I Ultra.

In the univariate Cox regression analysis, quartiles 2, 3, and 4 of the cTnI Sgx levels as well as detectable cTnI-Ultra levels were associated with the combined event and total mortality, readmission for myocardial infarction, and readmission for heart failure. In the multivariate analysis adjusted for clinical variables, both the cTnI Sgx and cTnI-Ultra levels remained predictive of the analysed cardiovascular events (Table 3). Although the detectable cTnI-Ultra showed comparable HR with quartile 2 cTnI Sgx, the association was more robust among the higher quartiles. Higher quartiles (3 or 4) demonstrated higher HR and adjusted HR for all the outcome measures. Cumulative survival curves according to quartiles of cTnI Sgx and to detectable cTnI- Ultra are shown in Figure 2.

**Table 3:** Risk of composite endpoint (mortality, myocardial infarction, and heart failure) according to quartiles of cTnI Sgx and detectable cTnI-ultra levels.

		cTnI Sgx			Detectable cTnI-Ultra
	Q_1_ (*n* = 207)	Q_2_ (*n* = 211)	Q_3_ (*n* = 210)	Q_4_ (*n* = 210)	(*n* = 344)
Five-year follow-up events among survivors					
Combined event (mortality, readmission for MI, and readmission for HF)					
Non-adjusted HR (95% CI)	1 (Ref)	3.88 (1.86–8.11)	9.57 (4.79–19.12)	14.88 (7.52–29.43)	3.65 (2.75–4.85)
*P*-value		*P* < 0.001	*P* < 0.001	*P* < 0.001	*P* < 0.001
Adjusted HR (95% CI)	1 (Ref)	1.87 (0.88–3.97)	3.32 (1.60–6.88)	4.19 (2.00–8.77)	1.78 (1.31–2.42)
*P*-value		*P* = 0.106	*P* = 0.001	*P* < 0.001	*P* < 0.001
All-cause mortality					
Non-adjusted HR (95% CI)	1 (Ref)	2.83 (1.26–6.36)	6.67 (3.16–14.07)	8.50 (4.07–17.79)	2.71 (1.92–3.82)
*P*-value		*P* = 0.012	*P* < 0.001	*P* < 0.001	*P* < 0.001
Adjusted HR (95% CI)	1 (Ref)	1.26 (0.55–2.90)	2.13 (0.96–4.73)	2.02 (0.89–4.58)	1.28 (0.88–1.86)
*P*-value		*P* = 0.586	*P* = 0.063	*P* = 0.095	*P* = 0.200
Readmission for MI					
Non-adjusted HR (95% CI)	1 (Ref)	9.00 (1.14–70.94)	12.00 (1.56–92.1)	17.35 (2.31–130.33)	2.94 (1.51–5.71)
*P*-value		*P* = 0.037	*P* = 0.017	*P* = 0.006	*P* = 0.002
Adjusted HR (95% CI)	1 (Ref)	6.04 (0.70–52.80)	6.76 (0.70–65.34)	9.44 (0.86–103.21)	1.93 (0.86–4.31)
*P*-value		*P* = 0.104	*P* = 0.099	*P* = 0.066	*P* = 0.109
Readmission for HF					
Non-adjusted HR (95% CI)	1 (Ref)	6.00 (0.72–49.75)	23.64 (3.20–174.68)	54.76 (7.57–396.20)	5.58 (3.26–9.54)
*P*-value		*P* = 0.097	*P* = 0.002	*P* < 0.001	*P* < 0.001
Adjusted HR (95% CI)	1 (Ref)	2.38 (0.27–20.64)	6.27 (0.77–51.17)	11.98 (1.42–100.72)	2.36 (1.28–4.35)
*P*-value		*P* = 0.433	*P* = 0.086	*P* = 0.022	*P* = 0.006

Q, quartile; CI, confidence interval; MI, myocardial infarction; HF, heart failure; HR, hazard ratio; Ref, reference; cTnI, cardiac troponin I. Variables in the multivariate Cox regression were adjusted for age, sex, history of MI, heart failure, arterial hypertension, diabetes, atrial fibrillation, and glomerular filtration rate. Detectable cTnI-Ultra levels were ≥6–39 ng/L, and the prevalence in the population was 41%.

The clinical variables selected for the multivariate analysis are shown in (Supplementary Table S7). To estimate the improvement in the prediction of mortality and readmission for both types of cTn (cTnI Sgx and cTnI-Ultra), a clinical model comprising age, sex, history of myocardial infarction, heart failure, hypertension, diabetes, atrial fibrillation, and kidney disease was designed, and its calibration and discrimination capacity were analysed using the C-index (Table 4) before and after adding both types of cTnI to the clinical model (Models A and B, respectively). In both models, the addition of cTnI Sgx or cTnI-Ultra significantly increased the prediction capability of cardiovascular events. However, there were no statistically significant differences between the two predictive models that included the aforementioned troponin assays.

**Table 4:** Discriminative efficiency of cTnI Sgx and detectable cTnI-ultra levels for clinical endpoints over clinical variables.

		Model A	Model B	*P*-value
	Clinical data	cTnI Sgx	Detectable cTnI-Ultra	Model A vs. B
Combined event (mortality, readmission for MI, readmission for HF) Harrell's C-index (95% CI)	0.77 (0.74–0.80)	0.79 (0.76–0.81)	0.78 (0.75–0.81)	0.323
*P*-value		*P* = 0.018	*P* = 0.036	
All-cause mortality Harrell's C-index (95% CI)	0.77 (0.74–0.80)	0.77 (0.74–0.81)	0.77 (0.74–0.81)	0.433
*P*-value		*P* = 0.292	*P* = 0.425	
Readmission for MI Harrell's C-index (95% CI)	0.77 (0.71–0.83)	0.79 (0.73–0.84)	0.78 (0.73–0.84)	0.871
*P*-value		*P* = 0.495	*P* = 0.466	
Readmission for HF Harrell's C-index (95% CI)	0.84 (0.79–0.87)	0.86 (0.83–0.89)	0.85 (0.81–0.89)	0.195
*P*-value		*P* = 0.062	*P* = 0.154	

Q, quartile; CI, confidence interval; MI, myocardial infarction; HF, heart failure; cTnI, cardiac troponin I; cTnI Sgx, cardiac troponin I Singulex; cTnI-Ultra, cardiac troponin I Ultra Siemens.

## 4 Discussion

Our study showed that ultra-sensitive cTnI (cTnI Sgx) levels and detectable contemporary cTnI (cTnI-Ultra) levels below the 99th percentile in patients treated in an emergency department were closely related to the cardiovascular risk profile. Clinical variables and cTnI levels were correlated with medium- and long-term cardiovascular events, and both analytical methods improved the predictive capacity without significant differences between them. Our study emphasizes the importance of considering cTnI levels close to the 99th percentile, using any analytical method, to identify patients at high risk of future cardiovascular events.

Studies on the predictive value of cTn levels below the 99th percentile have focused on subjects from the general population, chest pain evaluation to rule out myocardial ischaemia in stable patients, patients with stable chronic coronary artery disease, and patients treated in emergency departments for chest pain. In the general population, detectable cTn has been shown to be a significant predictor of cardiovascular events. cTnI appears to be a more specific marker of composite cardiovascular disease and coronary heart disease, whereas cTnT is more strongly associated with death from non-cardiovascular disease. Both cTnI and cTnT are associated with heart failure and death from cardiovascular disease (15, 16, 17, 18, 19, 20, 21, 22). In the Akershus Cardiac Examination 1950 Study, the cTnI level measured with a highly sensitive assay was predictive of the carotid atherosclerotic burden (23). In stable patients with chest pain evaluated using isotopic ergometry, the baseline cTnI Sgx level, even when only slightly elevated, was associated with induced ischaemia during stress testing (24). Similarly, a very slight elevation of the cTnI Sgx level in patients undergoing chest pain evaluation using isotopic stress testing was also associated with induced ischaemia (25). However, these tiny changes in the cTnI Sgx concentration during stress testing have not been demonstrated with other highly sensitive analytical methods for cTn measurement (26). In one study of patients with stable chronic heart disease, a risk gradient of cardiovascular death or heart failure based on the baseline cTnI Sgx concentration was demonstrated: patients with levels in the third tertile had higher risk, with a hazard ratio of 2.2 (95% confidence interval, 1.01–4.71), than patients in the first tertile (27).

Chest pain is one of the most common symptoms in patients admitted to emergency departments and the primary reason for which cTn measurement is clearly indicated. However, fewer than 10% of patients are diagnosed with ACS (28). The utility of a non-elevated cTn level in patients treated in emergency departments for chest pain lies primarily in ruling out myocardial infarction, both in protocols requiring two determinations (29) and in those recommending a single determination of cTn showing very low levels (30). However, in the medium and long term, detectable cTn confers an excess risk of cardiovascular events relative to undetectable cTn (9), especially if clinical variables are included in predictive models (10, 11). For patients in whom myocardial infarction has been ruled out, those with intermediate cTnI concentrations are more likely to have coronary artery disease as shown by coronary computed tomography angiography than those with low cTnI concentrations (31).

Our study provides additional information on patients treated in emergency departments with negative cTnI: those with levels close to the 99th percentile, especially if they have a clinical risk profile, have an excess risk of cardiovascular events in the medium and long term. In our study, we did not use traditional risk scales to predict cardiovascular events [e.g., Global Registry of Acute Coronary Events (GRACE) score and History, Electrocardiography, Age, Risk factors, Troponin (HEART) score]. These scales often require variables that are not consistently available in all emergency department medical records. The HEART score wich has been developed specifically for use in chest pain assessment in the emergency department can contribute to a better risk stratification, but it stem from before the high-sensitivity troponin era. Nevertheless, the GRACE score seems to be of limited value in low -risk patients such as those with normal hs-cTnT (10, 26, 27, 32, 33). In our study, all patients showed hemodynamic stability, no ischemic ECG changes and normal troponin, therefore, scores focused on clinical data could be expected to perform better. Therefore, we limited our analysis to clinical data that are invariably present in any emergency department medical record for a patient suspected to have ACS and for whom troponin measurement is requested. We assessed an analytical method for the determination of cTnI that can be considered ultra-sensitive, allowing the measurement of very small quantitative concentrations of cTnI much more accurately than contemporary or commonly marketed high-sensitivity methods (34). Garcia-Osuna et al. (13) studied the analytical characteristics of this highly sensitive cardiac biomarker, revealing that the ultra-sensitive method was approximately 10 times more sensitive than the currently used high-sensitivity cTnI method and that the proportion of healthy individuals with measurable cTn concentrations was 99.5%. Importantly, the healthy population in their study was selected based on strict clinical and analytical criteria. They also found that the median cTn level was significantly higher in men, patients of advanced age, and patients with impaired renal function (13). Certain biological and analytical factors can modify the cTn concentration without myocardial damage. In contemporary cTn measurement methods, as with any biochemical measurement, random errors or pre-analytical or analytical interferences may occur (35). In this regard, the cTnI Sgx assay did not detect significant cross-reactivity (34). Despite the advantages of this analytical method for the determination of cTn, we were unable to demonstrate that the predictive improvement for cardiovascular events in addition to the predictive capacity of clinical variables is significantly better than what can be obtained with other contemporary analytical methods. In this sense, the implementation of high-sensitivity cTn measurement has also shown no benefits over contemporary cTn measurement in terms of the short- and medium-term prognosis in patients diagnosed with myocardial infarction (36).

Identifying patients at high risk for cardiovascular events treated in emergency departments with negative cTn but levels close to the 99th percentile could have therapeutic implications. In the West of Scotland Coronary Prevention Study (WOSCOPS), the baseline cTn level was an independent predictor of myocardial infarction or death from coronary heart disease (37). The plasma cTnI levels were reduced by statin therapy, and reductions in cTnI concentrations were associated with better outcomes independent of low-density lipoprotein cholesterol lowering. In another study, empagliflozin, an inhibitor of the sodium–glucose cotransporter 2, was found to reduce the cTnI level in patients with diabetes (38). This could be one of the mechanisms underlying its beneficial effect in terms of preventing cardiovascular events. However, the current European clinical practice guidelines for cardiovascular prevention published in 2021 do not recommend the use of serum biomarkers such as natriuretic peptides or high-sensitivity cTn in patient risk stratification (39). Patients treated in emergency departments with cTn levels near the 99th percentile provide an excellent opportunity for cardiovascular risk stratification. In these cases, implementing measures such as statins for patients with hypercholesterolaemia or sodium–glucose cotransporter 2 inhibitors for those with diabetes is beneficial because both drugs have been shown to reduce cTn levels and improve the prognosis.

### 4.1 Limitations

This study had several limitations. First, this was a retrospective study conducted at a single hospital centre, and the clinician's decision to measure the cTnI level may have been influenced by the patients’ baseline characteristics. This may have resulted in selection of patients who were older and had a worse risk profile. Second, the variables collected for analysis were those present in medical records of patients treated in emergency departments for purely healthcare purposes. Therefore, essential variables affecting the prognosis may not have been adequately recorded, preventing their inclusion in the analysis. Third, we used an analytical method that is currently not available for healthcare purposes. However, the currently available highly sensitive analytical methods for cTn determination may be sufficient for adequate patient risk stratification because they allow detection of circulating cTn in more than 50% of patients with levels below the 99th percentile. Fourth, our population comprised patients of advanced age. It is possible that the 99th percentile used in our study to consider a non-elevated cTn level was excessively restrictive in these patients because the 99th percentile levels were considerably higher in studies of apparently healthy populations without restriction criteria (40). Fifth, models were both trained and tested on the same clinical dataset, to increase participant numbers and in the hope of statistical power. However, this poses a risk of over-fitting and type I error.

## 5 Conclusions

The herein-described approach using cTn to select patients for downstream investigation after myocardial infarction has been ruled out has major potential to improve patient outcomes. Regardless of the cTnI measurement method used, obtaining levels below the 99th percentile in patients treated in an emergency department allows the identification of patients with a worse cardiovascular risk profile and an adverse prognosis. Our study suggests that an integrated approach that combines predictive clinical variables, hemodynamic status, ECG findings and the measurement of cTnI can improve the prediction of outcomes in these patients.

## Data Availability

The original contributions presented in the study are included in the article/Supplementary Material, further inquiries can be directed to the corresponding author.
