# Supplementary material for: Prognostication in emergency room patients: comparing ultrasensitive and contemporary quantification of cardiac troponin levels below the 99th percentile
Source: Front Cardiovasc Med. 2025 Jan 13;11:1450619. doi: 10.3389/fcvm.2024.1450619 (PMC11769976; doi:10.3389/fcvm.2024.1450619)
Supplement: Supplementary file 1 [file Table1.docx]

Supplementary Material

**Supplementary Table S1.** Baseline characteristics according to quartiles of cTnI Sgx levels

|  | **Q_1_**  **(n = 207)** | **Q_2_**  **(n = 211)** | **Q_3_**  **(n = 210)** | **Q_4_**  **(n = 210)** | **P-value** |
| --- | --- | --- | --- | --- | --- |
| **Demographic variables** |  |  |  |  |  |
| Age, years | 48.1 ± 14.8 | 61.9 ± 14.6 | 69.0 ± 12.9 | 72.3 ± 12.7 | 0.001 |
| Female sex | 103 (49.8) | 86 (40.8) | 87 (41.4) | 78 (37.1) | 0.064 |
| **Clinical history** |  |  |  |  |  |
| Arterial hypertension | 49 (23.7) | 115 (54.5) | 140 (66.7) | 148 (70.48) | <0.001 |
| Diabetes | 17 (8.2) | 48 (22.7) | 54 (23.7) | 56 (26.7) | <0.001 |
| Current or previous smoker | 39 (18.8) | 46 (21.8) | 24 (11.4) | 25 (11.9) | 0.006 |
| Prior MI | 12 (5.8) | 37 (17.5) | 54 (27.1) | 69 (32.8) | <0.001 |
| Congestive heart failure | 4 (1.9) | 6 (2.8) | 9 (4.3) | 28 (13.3) | <0.001 |
| Peripheral arterial disease | 2 (1.0) | 6 (2.8) | 13 (6.2) | 17 (8.1) | 0.002 |
| Stroke or TIA | 0 (0.0) | 10 (4.7) | 19 (9.1) | 18 (8.6) | <0.001 |
| COPD | 20 (9.7) | 47 (22.3) | 38 (18.1) | 50 (23.8) | 0.001 |
| Charlson Index | 0 (0–1) | 1 (0–2) | 1 (0–2) | 2 (1–3) | <0.001 |
| **Symptoms** |  |  |  |  |  |
| Chest pain | 133 (64.3) | 134 (63.5) | 117 (55.7) | 91 (43.3) | <0.001 |
| Dyspnoea | 15 (7.3) | 23 (10.9) | 31 (14.8) | 44 (21.0) | <0.001 |
| Syncope | 7 (3.4) | 13 (6.2) | 10 (4.8) | 10 (4.8) | <0.001 |
| Other symptoms | 46 (22.2) | 41 (19.4) | 50 (23.8) | 66 (31.4) | 0.029 |
| **Vital signs** |  |  |  |  |  |
| SPB, mmHg | 131.5 ± 20.2 | 134.7 ± 23.4 | 140.3 ± 27.1 | 137.8 ± 27.5 | 0.002 |
| Heart rate, bpm | 83.7 ± 19.2 | 79.5 ± 20.7 | 80.6 ± 20.8 | 81.0 ± 23.6 | 0.030 |
| **Electrocardiogram** |  |  |  |  |  |
| Atrial fibrillation | 3 (1.5) | 16 (7.7) | 25 (11.9) | 47 (22.4) | <0.001 |
| LBBB or RBBB | 14 (6.8) | 15 (7.2) | 21 (10.0) | 28 (13.3) | 0.077 |
| Negative T wave | 9 (1.1) | 12(1.4) | 8(1.0) | 10(1.2) | 0.815 |
| ST-segment depression | 1(0.1) | 0(0.0) | 1(0.1) | 3(0.4) | 0.285 |
| **Laboratory tests** |  |  |  |  |  |
| eGFR, mL/min/1.73 m^2^ | 106.1 ± 30.5 | 97.5 ± 32.9 | 89 ± 32.6 | 79.8 ± 35.1 | <0.001 |
| Haemoglobin, g/dL | 13.5 ± 1.7 | 13.2 ± 1.9 | 12.9 ± 1.8 | 12.7 ± 2.1 | <0.001 |
| cTnI Sgx, ng/L | 0.72 ± 0.2 | 1.51 ± 0.3 | 2.7 ± 0.5 | 5.3 ± 1.3 | <0.001 |
| **Hospitalisation** | 32 (15.5) | 39 (18.5) | 49 (23.3) | 70 (33.3) | <0.001 |
| **In-hospital mortality** | 0 (0.0) | 2 (0.9) | 0 (0.0) | 3 (1.4) | 0.145 |

Data are presented as mean ± standard deviation, n (%), or median (interquartile range). Abbreviations: Q, quartile; MI, myocardial infarction; TIA, transient ischaemic attack; COPD, chronic obstructive pulmonary disease; SBP, systolic blood pressure; LBBB, left bundle branch block; RBBB, right bundle branch block; eGFR, estimated glomerular filtration rate (calculated by Modification of Diet in Renal Disease Study equation); cTnI Sgx, cardiac troponin I Singulex.

**Supplementary Table S2.** Main diagnoses according to quartiles of cTnI Sgx level

|  | **Q_1_**  **(n= 207)** | **Q_2_**  **(n = 211)** | **Q_3_**  **(n = 210)** | **Q_4_**  **(n = 210)** | **P-value** |
| --- | --- | --- | --- | --- | --- |
| **Chest pain** | 90 (43.5) | 93 (44.1) | 86 (41.0) | 42 (20.0) | <0.001 |
| **Congestive heart failure** | 3 (1.4) | 7 (3.3) | 9 (4.3) | 25 (11.9) | <0.001 |
| **Tachyarrhythmia** | 7 (3.4) | 10 (4.7) | 13 (6.2) | 25 (12.0) | 0.002 |
| **Respiratory infection** | 12 (5.8) | 19 (9.0) | 19 (9.0) | 14 (6.7) | 0.489 |
| **Gastrointestinal bleeding** | 1 (0.5) | 1 (0.5) | 0 (0.0) | 2 (1.0) | 0.571 |
| **Other gastrointestinal pathology** | 12 (5.8) | 14 (6.6) | 11 (5.2) | 11 (5.2) | 0.917 |
| **Syncope** | 4 (1.9) | 1 (0.5) | 7 (3.3) | 5 (2.4) | 0.212 |
| **Other infections** | 4 (1.9) | 2 (1.0) | 2 (1.0) | 5 (2.4) | 0.544 |
| **Sepsis** | 1 (0.5) | 1 (0.5) | 1 (0.5) | 4 (1.9) | 0.276 |
| **Bradycardia** | 0 (0.0) | 2 (1.0) | 1 (0.5) | 2 (1.0) | 0.536 |
| **Anaemia** | 0 (0.0) | 2 (1.0) | 1 (0.5) | 2 (1.0) | 0.536 |
| **Neurological disease** | 1 (0.5) | 2 (1.0) | 5 (2.4) | 2 (1.0) | 0.307 |
| **Neoplasia** | 1 (0.5) | 0 (0.0) | 0 (0.0) | 2 (1.0) | 0.298 |
| **Aortic disease** | 0 (0.0) | 0 (0.0) | 0 (0.0) | 2 (1.0) | 0.112 |
| **Hypertensive crisis** | 4 (1.9) | 5 (2.4) | 5 (2.4) | 1 (0.5) | 0.406 |
| **Renal failure** | 0 (0.0) | 1 (0.5) | 0 (0.0) | 1 (0.5) | 0.575 |
| **Other diagnoses** | 41 (19.8) | 42 (19.9) | 32 (15.2) | 49 (23.3) | 0.220 |

Data are presented as n (%). Abbreviations: Q, quartile; cTnI Sgx, cardiac troponin I Singulex.

**Supplementary Table S3.** Numbers and incidence rates (per 1000 person-years) of clinical endpoints according to quartiles of cTnI Sgx levels

|  |  | **Composite endpoint** | | | |
| --- | --- | --- | --- | --- | --- |
|  |  | **30-day** | **1-year** | **2-year** | **4-year** |
| **Quartile 1** | Number  Incidence rate | 0  0.00 | 1  0.40 | 3  0.61 | 8  0.82 |
| **Quartile 2** | Number  Incidence rate | 3  14.40 | 10  4.07 | 17  3.52 | 28  3.00 |
| **Quartile 3** | Number  Incidence rate | 4  19.18 | 19  7.93 | 33  7.19 | 56  6.55 |
| **Quartile 4** | Number  Incidence rate | 10  49.07 | 28  12.34 | 50  11.56 | 88  10.68 |
|  |  | **All-cause mortality** | | | |
|  |  | **30-day** | **1-year** | **2-year** | **4-year** |
| **Quartile 1** | Number  Incidence rate | 0  0.00 | 1  0.40 | 3  0.61 | 7  0.72 |
| **Quartile 2** | Number  Incidence rate | 3  14.40 | 8  3.24 | 13  2.66 | 21  2.21 |
| **Quartile 3** | Number  Incidence rate | 4  19.18 | 13  5.36 | 23  4.87 | 37  4.09 |
| **Quartile 4** | Number  Incidence rate | 6  29.23 | 15  6.26 | 26  5.56 | 49  5.59 |
|  |  | **Heart failure readmission** | | | |
|  |  | **30-day** | **1-year** | **2-year** | **4-year** |
| **Quartile 1** | Number  Incidence rate | 0  0.00 | 0  0.00 | 0  0.00 | 1  0.10 |
| **Quartile 2** | Number  Incidence rate | 0  0.00 | 0  0.00 | 2  0.41 | 4  0.42 |
| **Quartile 3** | Number  Incidence rate | 0  0.00 | 4  1.65 | 9  1.94 | 17  1.95 |
| **Quartile 4** | Number  Incidence rate | 4  19.62 | 12  5.22 | 21  4.76 | 37  4.60 |
|  |  | **Myocardial infarction readmission** | | | |
|  |  | **30-day** | **1-year** | **2-year** | **4-year** |
| **Quartile 1** | Number  Incidence rate | 0  0.00 | 0  0.00 | 0  0.00 | 1  0.10 |
| **Quartile 2** | Number  Incidence rate | 0  0.00 | 2  0.81 | 4  0.83 | 7  0.75 |
| **Quartile 3** | Number  Incidence rate | 0  0.00 | 3  1.25 | 4  0.86 | 9  1.02 |
| **Quartile 4** | Number  Incidence rate | 0  0.00 | 3  1.27 | 9  1.97 | 16  1.89 |

**Supplementary Table S4.** Numbers and incidence rates (per 1000 person-years) of clinical endpoints according to cTnI-Ultra levels

|  |  | **Composite endpoint** | | | |
| --- | --- | --- | --- | --- | --- |
|  |  | **30-day** | **1-year** | **2-year** | **4-year** |
| **Undetectable cTnI-Ultra** | Number | 2 | 12 | 28 | 59 |
|  | Incidence rate | 4.10 | 2.10 | 2.42 | 2.65 |
| **Detectable cTnI-Ultra** | Number | 15 | 46 | 75 | 116 |
|  | Incidence rate | 44.70 | 12.25 | 10.50 | 8.85 |
|  |  | **All-cause mortality** | | | |
|  |  | **30-day** | **1-year** | **2-year** | **4-year** |
| **Undetectable cTnI-Ultra** | Number | 2 | 9 | 22 | 44 |
|  | Incidence rate | 4.06 | 1.50 | 1.90 | 1.90 |
| **Detectable cTnI-Ultra** | Number | 11 | 28 | 43 | 70 |
|  | Incidence rate | 32.60 | 7.20 | 5.60 | 4.80 |
|  |  | **Heart failure readmission** | | | |
|  |  | **30-day** | **1-year** | **2-year** | **4-year** |
| **Undetectable cTnI-Ultra** | Number | 0 | 1 | 4 | 14 |
|  | Incidence rate | 0.00 | 0.20 | 0.30 | 0.62 |
| **Detectable cTnI-Ultra** | Number | 4 | 15 | 28 | 45 |
|  | Incidence rate | 11.92 | 3.90 | 3.80 | 3.30 |
|  |  | **Myocardial infarction readmission** | | | |
|  |  | **30-day** | **1-year** | **2-year** | **4-year** |
| **Undetectable cTnI-Ultra** | Number | 0 | 2 | 5 | 10 |
|  | Incidence rate | 0.00 | 0.34 | 0.43 | 0.40 |
| **Detectable cTnI-Ultra** | Number | 0 | 6 | 12 | 23 |
|  | Incidence rate | 0.00 | 1.60 | 1.60 | 1.60 |

**Supplementary Table S5.** Baseline characteristics according to cTnI-Ultra levels

|  | **Undetectable cTnI**  **(n = 494)** | **Detectable cTnI**  **(n = 344)** | **P-value** |
| --- | --- | --- | --- |
| **Demographic variables** |  |  |  |
| Age, years | 57.1 ± 16.6 | 71.2 ± 12.6 | <0.001 |
| Female sex | 211 (25.2) | 143 (17.1) | 0.742 |
| **Clinical history** |  |  |  |
| Arterial hypertension | 212 (25.3) | 240 (28.6) | <0.001 |
| Diabetes | 76 (9.0) | 99 (11.8) | <0.001 |
| Current or previous smoker | 93 (11.1) | 41 (4.9) | 0.007 |
| Prior MI | 75 (9.0) | 97 (11.6) | <0.001 |
| Congestive heart failure | 15 (1.8) | 32 (3.8) | <0.001 |
| Peripheral arterial disease | 15 (1.8) | 23 (2.7) | 0.012 |
| Stroke or TIA | 14 (1.7) | 33 (3.9) | <0.001 |
| COPD | 81 (9.7) | 74 (8.8) | 0.061 |
| Charlson Index | 1 (0–1) | 2 (0–2) | <0.001 |
| **Symptoms** |  |  |  |
| Chest pain | 312 (37.2) | 163 (19.5) | <0.001 |
| Dyspnoea | 51 (6.1) | 62 (7.4) | 0.001 |
| Syncope | 24 (2.9) | 16 (1.9) | 0.890 |
| Other symptoms | 102 (12.2) | 101 (12.0) | 0.004 |
| **Vital signs** |  |  |  |
| SPB, mmHg | 80.7 ± 21.2 | 81.9 ± 22.8 | 0.743 |
| Heart rate, bpm | 134.7 ± 22.8 | 138.1 ± 27.6 | 0.028 |
| **Electrocardiogram** |  |  |  |
| Atrial fibrillation | 26 (3.1) | 65 (7.8) | <0.001 |
| LBBB or RBBB | 32 (3.9) | 46 (5.5) | <0.001 |
| **Laboratory tests** |  |  |  |
| eGFR, mL/min/1.73 m^2^ | 99.6 ± 31.6 | 83.1 ± 35.4 | <0.001 |
| Haemoglobin, g/dL | 13.3 ± 1.7 | 12.7 ± 2.0 | <0.001 |
| cTnI-Ultra, ng/L |  | 1.6 ± 0.0 |  |
| **Hospitalisation** | 80 (9.6) | 110 (13.1) | <0.001 |
| **In-hospital mortality** | 1 (0.1) | 4 (0.5) | <0.001 |

Data are presented as mean ± standard deviation, n (%), or median (interquartile range). Abbreviations : MI, myocardial infarction; TIA, transient ischaemic attack; COPD, chronic obstructive pulmonary disease; SBP, systolic blood pressure; LBBB, left bundle branch block; RBBB, right bundle branch block; eGFR, estimated glomerular filtration rate (calculated by Modification of Diet in Renal Disease Study equation). Detectable cTnI-Ultra levels were ≥6 to 39 ng/L, and the prevalence in the population was 41%. Undetectable cTnI-Ultra levels were ≤6 ng/L, and the prevalence in the population was 59%.

**Supplementary Table S6.** Main diagnoses of hospitalised patients according to detectable cTnI-Ultra levels

|  | **Undetectable**  **cTnI-Ultra**  **(n = 494)** | **Detectable**  **cTnI-Ultra**  **(n = 344)** | **P-value** |
| --- | --- | --- | --- |
| **Chest pain** | 216 (25.8) | 95 (11.3) | <0.001 |
| **Congestive heart failure** | 15 (1.8) | 29 (3.5) | 0.001 |
| **Tachyarrhythmia** | 19 (2.3) | 36 (4.3) | <0.001 |
| **Respiratory infection** | 34 (6.9) | 30 (3.6) | 0.324 |
| **Gastrointestinal bleeding** | 2 (0.2) | 2 (0.2) | 0.715 |
| **Other gastrointestinal pathology** | 31 (3.7) | 17 (2.0) | 0.414 |
| **Syncope** | 9 (1.1) | 8 (1.0) | 0.611 |
| **Other infections** | 7 (0.8) | 6 (0.7) | 0.706 |
| **Sepsis** | 2 (0.2) | 5 (0.6) | 0.101 |
| **Bradycardia** | 1 (0.1) | 4 (0.5) | 0.076 |
| **Anaemia** | 2 (0.2) | 3 (0.4) | 0.388 |
| **Neurological disease** | 4 (0.5) | 6 (0.7) | 0.220 |
| **Neoplasia** | 1 (0.1) | 2 (0.2) | 0.366 |
| **Aortic disease** | 0 (0.0) | 2 (0.2) | 0.090 |
| **Hypertensive crisis** | 11 (1.3) | 4 (0.5) | 0.253 |
| **Renal failure** | 1 (0.1) | 1 (0.1) | 0.797 |
| **Other diagnosis** | 94 (11.2) | 70 (8.4) | 0.636 |

Data are presented as n (%).Detectable cTnI-Ultra levels were ≥6 to 39 ng/L, and the prevalence in the population was 41%.Undetectable cTnI-Ultra levels were ≤6 ng/L, and the prevalence in the populations was 59%.

**Supplementary Table S7.** Univariate Cox regression analyses for the clinical endpoint of the study

|  | **Univariate HR**  **(95% CI)** | **P-value** |
| --- | --- | --- |
| **Composite endpoint** |  |  |
| Age | 1.07 (1.05–1.08) | <0.001 |
| Sex | 1.20 (0.90–1.57) | 0.217 |
| Prior MI | 1.41 (1.04–1.90) | 0.025 |
| Congestive heart failure | 1.17 (0.72–1.88) | 0.526 |
| Arterial hypertension | 0.84 (0.61–1.15) | 0.279 |
| Diabetes | 1.56 (1.16–2.11) | 0.003 |
| Atrial fibrillation | 1.98 (1.42–2.75) | <0.001 |
| eGFR | 1.00 (1.00–1.00) | 0.855 |
| **All-cause mortality** |  |  |
| Age | 1.08 (1.06–1.10) | <0.001 |
| Sex | 1.51 (1.06–2.14) | 0.023 |
| Prior MI | 1.18 (0.80–1.73) | 0.401 |
| Congestive heart failure | 1.20 (0.66–2.18) | 0.547 |
| Arterial hypertension | 0.62 (0.42–0.91) | 0.014 |
| Diabetes | 1.27 (0.86–1.85) | 0.226 |
| Atrial fibrillation | 1.54 (1.02–2.33) | 0.041 |
| eGFR | 1.00 (1.00–1.00) | 0.663 |
| **Readmission for MI** | |  |
| Age | 1.02 (1.00–1.05) | 0.052 |
| Sex | 1.27 (0.66–2.47) | 0.474 |
| Prior MI | 2.32 (1.08–4.99) | 0.031 |
| Congestive heart failure | 0.39 (0.05–3.04) | 0.370 |
| Arterial hypertension | 1.00 (0.42–2.39) | 0.370 |
| Diabetes | 2.63 (1.30–5.33) | 0.007 |
| Atrial fibrillation | 1.25 (0.53–2.98) | 0.616 |
| eGFR | 1.00 (0.99–1.00) | 0.793 |
| **Readmission for HF** |  |  |
| Age | 1.07 (1.04–1.09) | <0.001 |
| Sex | 0.89 (0.56–1.41) | 0.622 |
| Prior MI | 1.66 (1.00–2.75) | 0.048 |
| Congestive heart failure | 1.48 (0.71–3.06) | 0.297 |
| Arterial hypertension | 1.92 (0.99–3.71) | 0.054 |
| Diabetes | 1.49 (0.90–2.49) | 0.123 |
| Atrial fibrillation | 2.21 (1.27–3.84) | 0.005 |
| eGFR | 1.00 (1.00–1.00) | 0.777 |

Abbreviations: CI, confidence interval; HR, hazard ratio; MI, myocardial infarction; HF, heart failure; eGFR, estimated glomerular filtration rate.
